# Stereotactic Cavity Irradiation or Whole-Brain Radiotherapy Following Brain Metastases Resection—Outcome, Prognostic Factors, and Recurrence Patterns

**DOI:** 10.3389/fonc.2020.00693

**Published:** 2020-05-08

**Authors:** Rami A. El Shafie, Thorsten Dresel, Dorothea Weber, Daniela Schmitt, Kristin Lang, Laila König, Simon Höne, Tobias Forster, Bastian von Nettelbladt, Tanja Eichkorn, Sebastian Adeberg, Jürgen Debus, Stefan Rieken, Denise Bernhardt

**Affiliations:** ^1^Department of Radiation Oncology, Heidelberg University Hospital, Heidelberg, Germany; ^2^National Center for Radiation Oncology (NCRO), Heidelberg Institute for Radiation Oncology (HIRO), Heidelberg, Germany; ^3^National Center for Tumor Diseases (NCT), Heidelberg, Germany; ^4^Institute of Medical Biometry and Informatics (IMBI), Heidelberg University Hospital, Heidelberg, Germany; ^5^Clinical Cooperation Unit Radiation Oncology (E050), German Cancer Research Center (dkfz), Heidelberg, Germany; ^6^Deutsches Konsortium Für Translationale Krebsforschung (DKTK), Partner Site Heidelberg, German Cancer Research Center (dkfz), Heidelberg, Germany; ^7^Heidelberger Ionenstrahltherapie-Zentrum (HIT), Heidelberg, Germany; ^8^Department of Radiation Oncology, University Medical Center Göttingen, Göttingen, Germany

**Keywords:** resection cavity, radiosurgery, palliative, radiotherapy, whole-brain radiotherapy, stereotactic, linear accelerator, robotic radiosurgery

## Abstract

**Introduction:** Following the resection of brain metastases (BM), whole-brain radiotherapy (WBRT) is a long-established standard of care. Its position was recently challenged by the less toxic single-session radiosurgery (SRS) or fractionated stereotactic radiotherapy (FSRT) of the resection cavity, reducing dose exposure of the healthy brain.

**Patients and Methods:** We analyzed 101 patients treated with either SRS/FSRT (*n* = 50) or WBRT (*n* = 51) following BM resection over a 5-year period. Propensity score adjustment was done for age, total number of BM, timepoint of BM diagnosis, controlled primary and extracranial metastases. A Cox Proportional Hazards model with univariate and multivariate analysis was fitted for overall survival (OS), local control (LC) and distant brain control (DBC).

**Results:** Median patient age was 61 (interquartile range, IQR: 56–67) years and the most common histology was non-small cell lung cancer, followed by breast cancer. 38% of the patients had additional unresected BM. Twenty-four patients received SRS, 26 patients received FSRT and 51 patients received WBRT. Median OS in the SRS/FSRT subgroup was not reached (IQR NA−16.7 months) vs. 12.6 months (IQR 21.3–4.4) in the WBRT subgroup (hazard ratio, HR 3.3, 95%-CI: [1.5; 7.2] *p* < 0.002). Twelve-months LC-probability was 94.9% (95%-CI: [88.3; 100.0]) in the SRS subgroup vs. 81.7% (95%-CI: [66.6; 100.0]) in the WBRT subgroup (HR 0.2, 95%-CI: [0.01; 0.9] *p* = 0.037). Twelve-months DBC-probabilities were 65.0% (95%-CI: [50.8; 83.0]) and 58.8% (95%-CI: [42.9; 80.7]), respectively (HR 1.4, 95%-CI: [0.7; 2.7] *p* = 0.401). In propensity score-adjusted multivariate analysis, incomplete resection negatively impacted OS (HR 3.9, 95%-CI: [2.0;7.4], *p* < 0.001) and LC (HR 5.4, 95%-CI: [1.3; 21.9], *p* = 0.018). Excellent clinical performance (HR 0.4, 95%-CI: [0.2; 0.9], *p* = 0.030) and better graded prognostic assessment (GPA) score (HR 0.4, 95%-CI: [0.2; 1.0], *p* = 0.040) were prognostic of superior OS. A higher number of BM was associated with a greater risk of developing new distant BM (HR 5.6, 95%-CI: [1.0; 30.4], *p* = 0.048). In subgroup analysis, larger cavity volume (HR 1.1, 95%-CI: [1.0; 1.3], *p* = 0.033) and incomplete resection (HR 12.0, 95%-CI: [1.2; 118.3], *p* = 0.033) were associated with inferior LC following SRS/FSRT.

**Conclusion:** This is the first propensity score-adjusted direct comparison of SRS/FSRT and WBRT following the resection of BM. Patients receiving SRS/FSRT showed longer OS and LC compared to WBRT. Future analyses will address the optimal choice of safety margin, dose and fractionation for postoperative stereotactic RT of the resection cavity.

## Introduction

Current international guidelines recommend neurosurgical extirpation for brain metastases (BM) that are either large and symptomatic or in cases where histologic confirmation is required (1, 2). For solitary BM, surgical resection can improve prognosis when compared to conventional irradiation alone (3). To avoid local recurrence, post-operative radiotherapy is recommended (4, 5). It can be delivered in the form of whole-brain radiotherapy (WBRT), which has proven effective in several phase-3 trials, although associated with substantial neurotoxicity (4–6). Alternatively, single-fraction stereotactic radiosurgery (SRS) or fractionated stereotactic radiotherapy (FSRT) of the resection cavity can be employed to avoid toxicity and preserve neurocognition (7, 8).

However, the use of resection cavity SRS comes at the cost of an increased risk of recurrence in the distant and untreated brain. Furthermore, the recent phase-3 trial by Brown et al. showed an increased risk of local recurrence at the irradiated cavity as well following SRS, as compared to WBRT (7, 8). Several reasons are discussed for the latter finding: The safety margin of 1–2 mm employed in the trials by Brown et al. (8) and Mahajan et al. (7) for radiosurgery were possibly too small to account for microscopic tumor infiltration. Also, for large cavities the dose of SRS was reduced down to 12 Gy—possibly an insufficient dose to achieve lasting tumor control (7, 8).

FSRT of the resection cavity has been discussed as a means to deliver more adequate doses to large cavities without increasing the risk of radionecrosis and has shown favorable local control rates in several retrospective analyses (9–11). It furthermore seems to be a feasible way of increasing the safety margins without risking a significant increase in toxicity (9, 12, 13).

Accounting for all the above, the increased risk of distant intracranial recurrence remains when omitting WBRT in favor of locally ablative treatment. New distant brain metastases can in turn have a detrimental effect on neurologic function, as well as survival (14, 15). Another risk following metastases resection is leptomeningeal spread, which in series with post-operative radiosurgery occurs in 15–30% of the cases and which risk WBRT can help reduce (16–21).

The current manuscript describes our single-center experience with 101 patients treated with post-operative SRS/FSRT or post-operative WBRT following BM resection. Propensity score adjustment was performed to account for imbalances in baseline covariates and reduce the risk of selection bias.

## Patients and Methods

One hundred one patients who received either SRS/FSRT (*n* = 50) or WBRT (*n* = 51) following BM resection between 2015 and 2019 were included in this analysis. Patient and treatment data was extracted from a clinical database maintained at our institution and from medical and official records (22). All reviews were performed following institutional guidelines and the Declaration of Helsinki of 1975 in its most recent version. Ethics approval for the study and a waiver of written informed consent was granted by the Heidelberg University ethics committee on April 12th, 2018 (#S-172/2018). Patient confidentiality was maintained by anonymizing patient data to remove any identifying information.

### Patient and Treatment Characteristics

Median patient age at the beginning of radiotherapy (RT) was 61 (interquartile range, IQR: 56–67) years and the most common histology was non-small cell lung cancer (NSCLC), followed by breast cancer. Thirty-eight percentage of the patients had additional unresected brain metastases that were either included in WBRT or treated with SRS for patients in the radiosurgery subgroup. For WBRT, systemic therapy was paused. Of the patients treated with radiosurgery, 11 (22%) received concomitant targeted therapies (e.g., immunotherapy, molecular therapy). Detailed patient characteristics are illustrated in Table 1. Macroscopic resection status was complete for 71 patients (70.3%) and incomplete for 30 patients (29.7%). RT was started within a median of 5.1 [IQR 3.9–7.0] weeks after the completion of surgery.

**Table 1:** Baseline characteristics.

	**SRS/FSRT (*n* = 50)**	**WBRT (*n* = 51)**	**Total (*n* = 101)**
**Age at radiotherapy (years)**			
Mean	59.1	62.1	60.6
Median	61	62	10.3
SD	11	9.3	61
Q1–Q3	54–65	56–70	56–67
Min.–Max.	29.0–80.0	36.0–78.0	29–80
**Gender**			
Female	30 [60%]	29	59 [58.42%]
Male	20 [40%]	22	42 [41.58%]
**Primary histology**			
NSCLC	22 [44.0%]	31 [60.8%]	53 [52.48%]
Breast cancer	10 [20.0%]	8 [15.7%]	18 [17.82%]
Gastrointestinal	6 [12.0%]	2 [3.9%]	8 [7.92%]
Melanoma	2 [4.0%]	5 [9.8%]	7 [6.93%]
Renal cell carcinoma	4 [8.0%]	2 [3.9%]	6 [5.94%]
Head and neck	0 [0.0%]	2 [3.9%]	2 [1.98%]
Gynecological	0 [0.0%]	1 [2.0%]	1 [0.99%]
Other	6 [12.0%]	0 [0.0%]	6 [5.94%]
**Additional BM**			
Absent	41 [82.0%]	22 [43.1%]	63 [62.38%]
Present	9 [18.0%]	29 [56.9%]	38 [37.62%]
**Total number of BM**			
Mean	1.2	2.8	2
Median	1	2	1
SD	0.6	2.2	1.8
Q1–Q3	1–2	1–5	1–2
Min.–Max.	1–4	1–9	1–9
**Time primary diagnosis until diagnosis of BM (months)**			
Mean	36.2	23.9	30
Median	21.1	7.8	14.7
SD	50	39.2	45.1
Q1–Q3	3.8–35.7	1.7–29.5	1.7–34.5
Min.–Max.	0.0–213.8	0.0–223.5	0–223.5
**Timepoint of BM diagnosis**			
Metachronous	39 [78.0%]	26 [51.0%]	65 [64.36%]
Synchronous	11 [22.0%]	25 [49.0%]	36 [35.64%]
**Primary tumor controlled**			
No	8 [16.0%]	25 [49.0%]	33 [32.67%]
Yes	42 [84.0%]	26 [51.0%]	68 [67.33%]
**Extracerebral metastases**			
No	35 [70.0%]	25 [49.0%]	60 [59.41%]
Yes	15 [30.0%]	26 [51.0%]	41 [40.59%]
**Resection status**			
Complete	39 [78.0%]	32 [62.7%]	71 [70.3%]
Incomplete	11 [22.0%]	19 [37.3%]	30 [29.7%]
**KPI at initial presentation for SRS (%)**			
≤70	12 [24.0%]	16 [31.3%]	18 [27.72%]
80	16 [32.0%]	20 [39.2%]	36 [35.64%]
≥90	22 [44.0%]	15 [29.5%]	36 [36.63%]
**Time surgery until RT (weeks)**			
Mean	6.2	5.5	5.8
Median	5.7	4.4	3.1
SD	2.3	3.7	5.1
Q1–Q3	4.4–7.3	2.7–6.6	3.9–7
Min.–Max.	1.7–12.4	1.4–16.6	1.4–16.6
**RPA class**			
1	17 [34.0%]	7 [13.7%]	24 [23.76%]
2	30 [60.0%]	37 [72.5%]	67 [66.34%]
3	3 [6.0%]	7 [13.7%]	10 [9.9%]
**GPA Score**			
1	37 [74.0%]	17 [33.3%]	54 [53.47%]
2	11 [22.0%]	23 [45.1%]	34 [33.66%]
3	2 [4.0%]	11 [21.6%]	13 [12.87%]

*SRS, stereotactic radiosurgery; WBRT, whole-brain radiotherapy; NSCLC, non-small cell lung cancer; SD, standard deviation; BM, brain metastases; KPI, Karnofsky performance scale index; RPA, recursive partitioning analysis; GPA, graded prognostic assessment*.

For all cranial RT, an individual head fixation mask was fitted for each patient. Delineation and treatment planning for SRS/FSRT was performed as previously described (23, 24). Target definition was based on high-resolution computed tomography (CT) and current magnetic resonance imaging (MRI). Standardized imaging protocols were used for all patients, complying to the following specifications: CT scan was acquired with 1 mm slice thickness. MRI contained a contrast-enhanced, T1-weighted, three-dimensional sequence with multiplanar reconstruction and a slice thickness of ≤ 1 mm. The MRI was thoroughly co-registered and served as basis for target and organs at risk (OAR) delineation. Cavity and residual tumor—if applicable—were contoured as clinical target volume (CTV). A safety margin of 1–3 mm was added by isotropic expansion and slightly adapted by an experienced physician when necessary to account for microscopic tumor spread. Technical uncertainties were accounted for by an additional margin of 1 mm, generating a planning target volume (PTV) so that typically a total margin of 2–4 mm around the visible cavity resulted. Prior to the publication of the phase-3 trial by Brown et al. (8), all patients were treated with FSRT following the institutional standard. Later on, the decision of SRS vs. FSRT was made on a case-by-case basis, considering all relevant medical aspects, as well as patient preference. Dose prescription for SRS was done in analogy to the phase-3 trial by Brown et al. and single doses between 12 and 20 Gy were administered depending on cavity volume (detailed in Table 2) (8). Dose prescription for FSRT was either 6 × 5, 7 × 5, or 5 × 6 Gy to the 70–89% isodose, covering at least 98% of the PTV. Historically, the regimen of 6 × 5 Gy was the institutional standard for FSRT. Later on this was adapted to 7 × 5 Gy following the promising data published for this regimen (9, 10). The regimen of 5 × 6 Gy was only utilized in one case only where a reduction of the number of fractions was required for organizational reasons related to other pending treatments. In cases with incomplete resection, no systematic special consideration was given to the residual tumor within the PTV in terms of dose prescription. Treatment planning was done in Accuray's Multiplan v5.3 and subsequent versions, while treatment was delivered using CyberKnife M6 (Accuray Inc., Sunnyvale, California). Details on target delineation and performed treatment are listed in Table 2.

**Table 2:** Treatment characteristics.

	**SRS/FSRT (*n* = 50)**	**WBRT (*n* = 51)**	**Total (*n* = 101)**
**Radiotherapy technique (*****n*** **=** **101)**			
FSRT	26 [52.0%]	0 [0.0%]	26 [25.74%]
SRS	24 [48.0%]	0 [0.0%]	24 [23.76%]
WBRT	0 [0.0%]	51 [100.0%]	51 [50.49%]
**Radiosurgery margin dose (Gy) (*****n*** **=** **50)**			
12	4 [8.0%]	– –	4 [3.96%]
14	5 [10.0%]	– –	5 [4.95%]
15	2 [4.0%]	– –	2 [1.98%]
16	6 [12.0%]	– –	6 [5.94%]
17	3 [6.0%]	– –	3 [2.97%]
18	5 [10.0%]	– –	5 [4.95%]
20	1 [2.0%]	– –	1 [0.99%]
30 (5 or 6 fractions)	11 [22.0%]	– –	62 [61.39%]
35 (7 fractions)	13 [26.0%]	– –	13 [12.87%]
**Number of fractions (*****n*** **=** **101)**			
1	26 [52.0%]	0 [0.0%]	26 [25.74%]
5	2 [4.0%]	0 [0.0%]	2 [1.98%]
6	9 [18.0%]	0 [0.0%]	9 [8.91%]
7	13 [26.0%]	0 [0.0%]	13 [12.87%]
10	0 [0.0%]	51 [100.0%]	5 [50.49%]
**BED**_**10**_ **(*****n*** **=** **101)**			
Mean	44.6	39	41.8
Median	45	39	39
SD	8.25	0	6.4
Q1–Q3	41.6–52.5	39–39	39–45
Min.–Max.	26.4–60.0	39.0–39.0	26.4–60
**Cavity volume (*****n*** **=** **50)**			
Mean	10.9	–	–
Median	7	–	–
SD	13.5	–	–
Q1–Q3	3.9–13	–	–
Min.–Max.	0.5–86.0	–	–
**PTV volume (*****n*** **=** **50)**			
Mean	22.4	–	–
Median	18.2	–	–
SD	19.8	–	–
Q1–Q3	9.6–28.1	–	–
Min.–Max.	0.8–126.5	–	–
**Number of additional BM treated with SRS (*****n*** **=** **50)**			
0	32 [64.0%]	–	32 [31.7%]
1	12 [24.0%]	–	12 [11.9%]
2	2 [4.0%]	–	2 [2%]
3	4 [8.0%]	–	4 [4%]

*SRS, stereotactic radiosurgery; FSRT, fractionated stereotactic radiotherapy; WBRT, whole-brain radiotherapy; BED, biologically equivalent dose; PTV, planning target volume; BM, brain metastases*.

Treatment planning for WBRT was performed using a 3 mm computed tomography (CT). The prescribed dose for WBRT was 30 Gy in 10 fractions. Treatment was delivered at a linear accelerator with two laterally opposing fields following three-dimensional conformal treatment planning for WBRT, as has been previously described (25, 26).

## Statistical Analysis

Descriptive statistics for baseline variables include means (standard deviation, SD) and/or median (IQR and range, as appropriate) for continuous variables and absolute and relative frequencies for categorical variables. The median and its corresponding quantiles of the follow-up time are calculated using the reverse Kaplan-Meier method (27). Overall survival (OS) is calculated from the beginning of RT to the date of death or last follow-up. Local control (LC) and distant brain control (DBC) are calculated from the beginning of RT to last imaging follow-up or confirmed progression. OS, LC and DBC are investigated using the method of Kaplan-Meier (KM). Additionally, a propensity score is calculated based on a logistic regression model for the treatment modality (SRS/FSRT vs. WBRT) including the prognostic covariates of age, time from primary diagnosis to diagnosis of BM, primary tumor control, extracranial metastases and total number of BM. To adjust for differences in the variables considered in the propensity score model, the score is used as covariate in multivariate Cox models for OS, LC, and DBC (28). To identify prognostic factors on those endpoints, univariate Proportional Cox regression models are calculated for all baseline characteristics listed in Tables 1, 2. Variables which seem to influence the outcome variable in univariate analysis and those of special clinical interest are considered in multivariate Cox regression. Since this is a retrospective exploratory data analysis, *p*-values are of descriptive nature. Statistical analyses are performed with the software *R Version 3.6.2*.

## Results

### Overall Survival

**Figure 1:**
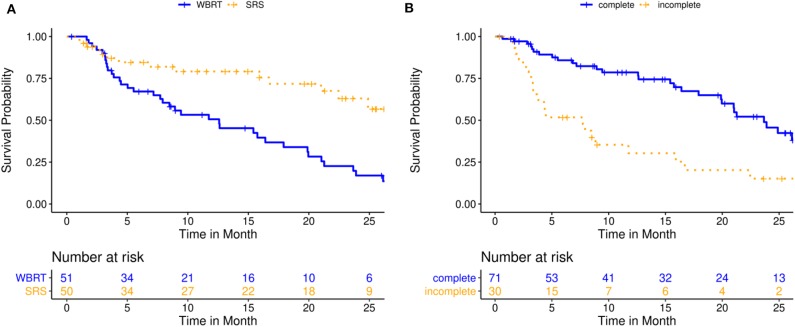
Overall survival for 101 patients treated with either post-operative single- or multisession stereotactic radiosurgery (SRS) or whole-brain radiotherapy (WBRT). **(A)** Kaplan-Meier curves stratified by treatment modality (*p* = 0.002); **(B)** Kaplan-Meier curves stratified by resection status (*p* < 0.001).

Median follow-up time for overall survival (OS) was 22.8 months (IQR: 9.0–31.9) for the entire cohort. At the time of this analysis, 86 patients had died, and 15 patients were still alive, corresponding to 63.7% survival probability at 12 months (KM estimate; 95%-CI: 54.2–74.7) and 36.0% at 24 months (KM estimate; 95%-CI: 25.8–50.2). Median OS was 19.9 months (IQR: NA−6.8) for the entire cohort. In univariate analysis, higher age (HR 1.1, 95%-CI: [1.0; 1.1], *p* = 0.040), higher number of BM (hazard ratio, HR 1.2, 95%-CI: [1.1; 1.4], *p* = 0.003), incomplete resection (HR 3.1, 95%-CI: [1.8; 5.6], *p* < 0.001), reduced clinical performance (Karnofsky performance index, KPI ≤ 70%) (HR 2.7, 95%-CI: [1.5; 4.9], *p* < 0.001), lung cancer histology (HR 2.6, 95%-CI: [1.4; 4.7], *p* = 0.001) and WBRT (HR 3.0, 95%-CI: [1.6; 5.5], *p* < 0.001) were associated with shorter OS, while a controlled primary (HR 0.5, 95%-CI: [0.3; 0.9], *p* = 0.029) and good clinical performance (KPI ≥ 90%) (HR 0.4, 95%-CI: [0.2; 0.8], *p* = 0.005) were prognostic of longer OS. Additionally, better recursive partitioning analysis (RPA) class (HR 0.4, 95%-CI: [0.2; 0.9], *p* = 0.023) and graded prognostic assessment (GPA) score (HR 0.3, 95%-CI: [0.2; 0.5], *p* < 0.001) were associated with superior OS. In propensity score-adjusted multivariate analysis, incomplete resection (HR 3.9, 95%-CI: [2.0; 7.4], *p* < 0.001) and WBRT (HR 3.3, 95%-CI: [1.5; 7.2], *p* = 0.002) were independently prognostic for inferior OS, while a KPI ≥ 90% (HR 0.4, 95%-CI: [0.2; 0.9], *p* = 0.030) and better GPA score (HR 0.4, 95%-CI: [0.2; 1.0], *p* = 0.040) were prognostic factors for superior OS. Detailed results on factors prognostic for OS are illustrated in Tables 3, 4 and Figure 1.

**Table 3:** Factors significant in univariate cox regression for the endpoints of overall survival, local control and distant brain control with corresponding hazard ratios and *p*-values.

	**HR**	**95% CI for HR**	***p***
**UNIVARIATE ANALYSIS FOR ENTIRE COHORT (*****n*** **=** **101)**			
**Overal survival**			
Age at radiotherapy	1.03	1.00–1.06	0.040
Additional BM present	1.93	1.10–3.39	0.021
Total number of BM	1.25	1.08–1.45	0.003
Timepoint of BM diagnosis	1.78	1.01–3.14	0.047
Controlled primary	0.53	0.30–0.94	0.029
Incomplete resection	3.15	1.78–5.55	<0.001
KPI ≤ 70%	2.73	1.51–4.94	0.001
KPI ≥90%	0.41	0.22–0.77	0.005
Lung cancer histology	2.59	1.45–4.65	0.001
Whole-brain radiotherapy	2.98	1.60–5.55	0.001
RPA class 1	0.41	0.19–0.89	0.023
RPA class 3	3.62	1.66–7.89	0.001
GPA score >2	0.29	0.16–0.53	<0.001
GPA score 1.5–2.0	2.04	1.14–3.67	0.017
GPA score 0–1	3.04	1.48–6.28	0.003
**Local control**			
Incomplete resection	3.80	1.05–13.77	0.042
**Distant brain control**			
Additional BM present	2.50	1.23–5.08	0.011
Total number of BM	1.27	1.06–1.52	0.009
**UNIVARIATE ANALYSIS FOR SRS/FSRT SUBGROUP (*****n*** **=** **50)**			
**Overal survival**			
Male gender	3.05	1.02–9.12	0.046
Incomplete resection	5.24	1.81–15.24	0.002
KPI ≤ 70%	4.53	1.55–13.27	0.006
Lung cancer histology	4.35	1.41–13.46	0.011
GPA score 0–1	4.67	1.02–21.46	0.047
**Local control**			
Incomplete resection	12.04	1.23–118.30	0.033
PTV volume	1.03	1.00–1.05	0.033
**Distant brain control**			
None	–	–	–

*SRS, stereotactic radiosurgery; FSRT, fractionated stereotactic radiotherapy; WBRT, whole-brain radiotherapy; SD, standard deviation; BM, brain metastases; KPI, Karnofsky performance scale index; RPA, recursive partitioning analysis; GPA, graded prognostic assessment; PTV, planning target volume*.

**Table 4:** Factors analyzed in propensity score-adjusted multivariate cox regression for the endpoints of overall survival, local control and distant brain control with corresponding hazard ratios and *p*-values.

	**HR**	**95% CI for HR**	***p***
**MULTIVARIATE ANALYSIS FOR ENTIRE COHORT (*****n*** **=** **101)**			
**Overal survival**			
Incomplete resection	3.85	2.00–7.44	<0.001
KPI ≤ 70%	1.11	0.52–2.39	0.784
KPI ≥90%	0.40	0.17–0.91	0.030
Lung cancer histology	1.86	0.86–4.02	0.113
WBRT	3.34	1.55–7.20	0.002
GPA score >2	0.41	0.18–0.96	0.040
RPA class 1	0.60	0.19–1.91	0.392
Propensity score	5.20	1.18–22.86	0.029
**Local control**			
SRS	0.18	0.04–0.90	0.037
Incomplete resection	5.39	1.33–21.89	0.018
Propensity score	6.42	0.47–87.14	0.162
**Distant brain control**			
Incomplete resection	1.82	0.85–3.90	0.126
≥ 3 lesions	5.56	1.01–30.49	0.048
Propensity score	2.68	0.24–29.40	0.419
**MULTIVARIATE ANALYSIS FOR SRS/FSRT SUBGROUP (*****n*** **=** **50)**			
**Overal survival**			
Incomplete resection	5.09	1.63–15.92	0.005
KPI ≤ 70%	4.32	1.29–14.41	0.017
Lung cancer histology	1.99	0.56–7.063	0.288
**Local control**			
Incomplete resection	8.35	0.70–99.98	0.094
PTV volume	1.06	0.93–1.21	0.360
**Distant brain control**			
None	–	–	–

*SRS, stereotactic radiosurgery; FSRT, fractionated stereotactic radiotherapy; WBRT, whole-brain radiotherapy; SD, standard deviation; BM, brain metastases; KPI, Karnofsky performance scale index; RPA, recursive partitioning analysis; GPA, graded prognostic assessment; PTV, planning target volume*.

### Local Control

**Figure 2:**
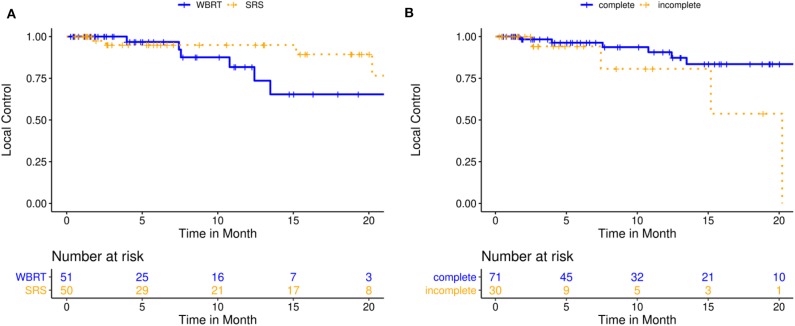
Local control for 101 patients treated with either post-operative single- or multisession stereotactic radiosurgery (SRS) or whole-brain radiotherapy (WBRT). **(A)** Kaplan-Meier curves stratified by treatment modality (*p* = 0.227); **(B)** Kaplan-Meier curves stratified by resection status (*p* = 0.018).

Median follow-up time for cavity local control (LC) and distant brain control (DBC) was 8.6 months (IQR: 15.9–3.0) for the entire cohort. At the time of this analysis, 10 patients had relapsed at the resection cavity, corresponding to 88.7% LC probability at 12 months (KM estimate; 95%-CI: 80.3–98.0) and 71.0% at 24 months (KM estimate; 95%-CI: 54.4–92.6). Median LC was not reached for the entire cohort (IQR: NA−20.2 months). In univariate analysis, incomplete resection (HR 3.8, 95%-CI: [1.1; 13.8], *p* = 0.042) was associated with inferior LC. In propensity score-adjusted multivariate analysis, both stereotactic treatment (compared to WBRT) (HR 0.2, 95%-CI: [0.04; 0.9], *p* = 0.037) and incomplete resection (HR 5.4, 95%-CI: [1.3; 21.9], *p* = 0.018) were independently prognostic of superior and inferior LC, respectively. Detailed results on factors prognostic for LC are illustrated in Tables 3, 4 and Figure 2.

### Distant Brain Control

**Figure 3:**
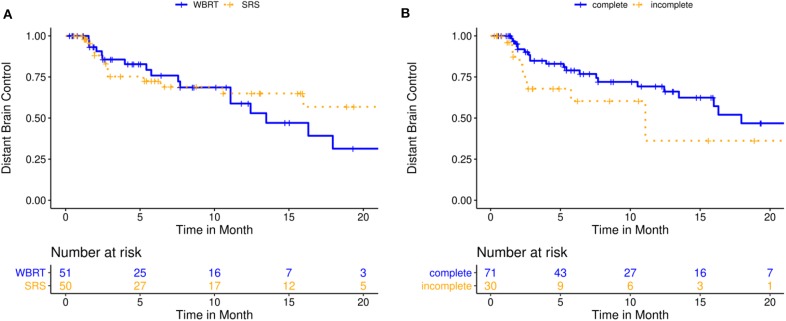
Distant brain control for 101 patients treated with either post-operative single- or multisession stereotactic radiosurgery (SRS) or whole-brain radiotherapy (WBRT). **(A)** Kaplan-Meier curves stratified by treatment modality (*p* = 0.401); **(B)** Kaplan-Meier curves stratified by resection status (*p* = 0.126).

At the time of this analysis, 32 patients had developed new BM in the distant brain, corresponding to 62.0% DBC probability at 12 months (KM estimate; 95%-CI: 50.9–75.5) and 35.6% at 24 months (KM estimate; 95%-CI: 20.2–62.8). Median DBC was 16.3 months for the entire cohort (IQR: NA−5.75 months). A higher total number of BM was associated with a greater risk of developing new distant BM in univariate (HR 1.3, 95%-CI: [1.1; 1.5], *p* = 0.009) and propensity score-adjusted multivariate analysis (HR 5.6, 95%-CI: [1.0; 30.4], *p* = 0.048). Detailed results on factors prognostic for DBC are illustrated in Tables 3, 4 and Figure 3.

### Subgroup Analysis

**Figure 4:**
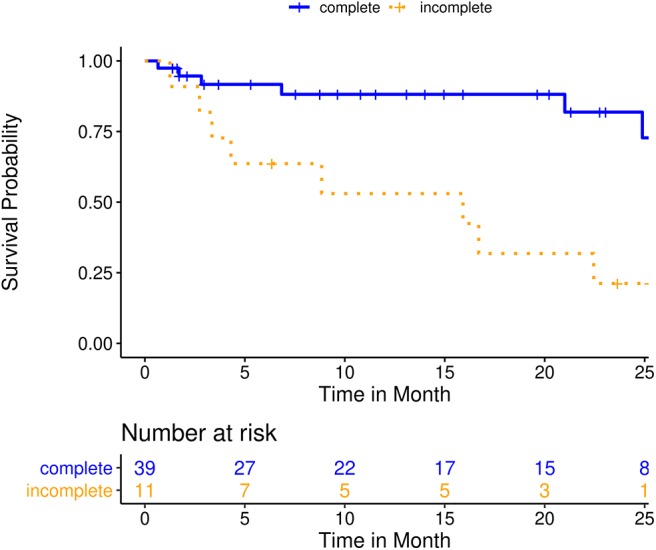
Overall survival for a subgroup analysis of 51 patients treated with post-operative single- or multisession stereotactic radiosurgery (SRS); Kaplan-Meier curves stratified by resection status (*p* < 0.005).

A subgroup analysis of the patients treated with SRS/FSRT was performed to evaluate the impact of treatment-specific parameters on the outcome. Median OS, LC and DBC in this subgroup were not reached. Kaplan-Meier estimate for survival probability was 79.1% (95%-CI: 67.8–92.4) at 12 months and 63.0% (95%-CI: 47.9–82.9) at 24 months. Kaplan-Meier estimate for LC probability was 94.9% (95%-CI: 88.3–100.0) at 12 months and 76.6% (95%-CI: 54.9–100.0) at 24 months. Kaplan-Meier estimate for DBC was 65.0% (95%-CI: 50.8–83.0) at 12 months and 56.9% (95%-CI: 39.7–81.4) at 24 months. Incomplete resection (HR 5.2, 95%-CI: [1.8; 15.2], *p* = 0.002), a KPI ≤ 70% (HR 4.5, 95%-CI: [1.5; 13.3], *p* = 0.006), lung cancer histology (HR 4.3, 95%-CI: [1.4; 13.5], *p* = 0.011) and low GPA score (HR 4.7, 95%-CI: [1.0; 21.5], *p* = 0.047) were associated with inferior OS in univariate analysis. Incomplete resection and KPI ≤ 70% stayed prognostic of inferior OS in multivariate analysis. Larger cavity PTV volume (HR 1.1, 95%-CI: [1.0; 1.3], *p* = 0.033) and incomplete resection (HR 12.0, 95%-CI: [1.2; 118.3], *p* = 0.033) were associated with inferior local control in univariate analysis. No additional treatment-specific parameters with noticeable impact on DBC could be identified. Four patients (8%) relapsed in the form of leptomeningeal spread during follow-up. Three patients (6%) developed radionecrosis at the irradiated cavity. All of those patients had been treated with single-session SRS at doses of 12, 15, and 18 Gy, respectively. Detailed results of the subgroup analysis are displayed in Tables 3, 4 and Figure 4. Of the patients with incomplete resection, 11 (36.7%) were in the radiosurgery subgroup. Three of the incompletely resected patients developed local failure, one of those in the form of leptomeningeal spread. Those cases comprised three of the total of four local failures in the SRS subgroup.

## Discussion

We performed a propensity score-adjusted comparison of SRS/FSRT and WBRT following the resection of brain metastases. In the SRS subgroup, patients showed longer OS and LC compared to the WBRT subgroup. The extent of macroscopic resection was the strongest prognostic factor across subgroups for local control and survival. Additional favorable prognosticators included good clinical performance and better RPA and GPA scores; unfavorable prognosticators included lung cancer histology.

The role of postoperative WBRT in improving local and intracranial control following the resection of BM has been demonstrated in the phase-3 EORTC trial by Kocher et al. (5). However, several trials have demonstrated the detrimental effect of WBRT on neurocognition and quality of life (8, 14, 15). Aiming to reduce treatment toxicity, SRS of the resection cavity has emerged as an alternative to WBRT: Early series demonstrated 12-months LC rates of up to 79% and showed that the addition of a 2 mm margin around the cavity lowered the risk of local relapse from 16 to 3% (29). Nevertheless, the recently published phase-3 trial by Brown et al. showed significantly inferior cavity local control following SRS, compared to WBRT, posing unanswered questions regarding the optimal margin width, dose and fractionation of postoperative cavity SRS (8).

In our analysis, we found the Kaplan-Meier estimate for local failure probability at 12 months to be 5% following SRS/FSRT and 18% following WBRT. Twent-four months after RT, local failure probability was 23% following SRS/FSRT cases and 34% of the WBRT cases. In all cases, a margin of 2–4 mm was utilized for radiosurgery treatment planning and 52% of the stereotactic cases were treated with multifraction FSRT. Our results regarding local control compare favorably to those of the trials by Brown et al. and Mahajan et al., where 12-months cavity local control rates following SRS were 60.5 and 72%, respectively (7, 8). In this regard, our results are in agreement with a number of series that demonstrated that the use of fractionated regimens could lead to improved LC (9–11). While in single-session SRS, prescription dose is reduced for large cavities to avoid toxicity, FSRT allows for the delivery of a higher biologically effective dose (BED) with a higher probability of tumor control (11, 30). In the subgroup analysis we performed for patients treated with SRS/FSRT, we could not identify FSRT vs. SRS or the BED as significant factors influencing local control. However, this could possibly be attributed to the small number of local failure events (*n* = 4) and generally favorable LC in this cohort.

We could identify the resection status as a strong prognostic factor for OS, as well as local control at the cavity. This finding was consistent between the analysis of the entire cohort, as well as the subgroup analysis considering only patients who received SRS/FSRT of the resection cavity. It emphasizes the importance of complete macroscopic resection, ideally confirmed by intraoperative or early post-operative MR-imaging for the patient's overall prognosis. It furthermore suggests that irradiation doses typically applied within the context of post-operative RT (WBRT or reduced-dose cavity radiosurgery) are insufficient to lastingly control macroscopic tumor (11, 31). Thus, if incomplete resection is unavoidable, it could possibly improve overall prognosis to treat the residual macroscopic tumor with a higher dose than the cavity PTV (e.g., in the form of an integrated boost), which however was not systematically done for the patients in this analysis.

Another danger posed by incomplete resection is the increased risk of leptomeningeal spread (LMD), which when it occurs, is severely detrimental for overall prognosis (32, 33). Besides the risk of iatrogenic tumor spread during neurosurgical resection, the early re-growth and potential meningeal spread of residual macroscopic tumor is a serious risk, leading to early local recurrence (34–36). It is facilitated by the specific pro-proliferatory cavity microenvironment, combined with a deficient blood-brain barrier, as has been demonstrated following the resection of malignant gliomas (37). The risk of post-operative LMD has been quantified to between 11% and up to 24% in 2 years in several retrospective series for patients receiving post-operative SRS. Here, breast cancer histology was identified as a significant risk factor (17–19, 21). Johnson et al. described it to be significantly higher for post-operative SRS than for post-operative WBRT (16.9 vs. 5.2% *p* < 0.01) (17). In our series, four (8%) of the patients in the SRS subgroup developed LMD at a median imaging follow-up of 8.6 months, which compares favorably to the figures found in literature.

The timely beginning of post-operative RT is another important factor for avoiding local relapse (38). Although in our current analysis, the length of the interval between resection and RT was not significantly associated with prognosis, it can nevertheless have relevant implications for correct treatment planning in the context of stereotactic RT: Several and partly contradicting findings have been published regarding the geometric and volumetric changes of the resection cavity over time, which heighten the uncertainties of correct target delineation for post-operative SRS/FSRT (36, 39). In an earlier analysis, we observed a median cavity size of 6.96 ccm, which was significantly smaller than the unresected lesion at 8.71 ccm (*p* = 0.019). Furthermore, we found a similarity coefficient of only 53% between the volumes of cavity and unresected lesion, indicating a small degree of anatomical overlap. The explanation for this finding lies in relevant changes of configuration and potential location shifts that can be observed for post-operative cavities (23).

One way of addressing the abovementioned uncertainties in target volume delineation is by increasing the width of the safety margin added to the visible cavity. The consensus contouring guidelines for cavity SRS published by Soliman et al. in the wake of the phase-3 trials by Brown et al. (8) and Mahajan et al. (7) describe a high level of overall agreement for the delineation of the cavity clinical target volume (40). However, the extent of additional safety margin, as well as the question of including the complete surgical tract and associated meningeal enhancement into the radiosurgical target volume are subject of frequent discussion (31, 40, 41). For single-session SRS, the prescription dose must be reduced with increasing target size to avoid an increasing risk of radionecrosis, but potentially compromising local control in turn, as discussed earlier (7, 8, 42, 43). In a previous systematic dosimetric analysis, we could demonstrate that for a fractionated regimen of 7 x 5 Gy, the use of a total margin of 4 mm (3 mm CTV, 1 mm PTV) is feasible, including the entire surgical tract and simultaneously delivering an escalated BED to the target volume (23). Notably in our present analysis, none of the patients receiving fractionated stereotactic regimens (48%), and particularly none of those treated with 7 × 5 Gy (26%) developed signs of radiation necrosis.

Combining the arguments and results discussed above with the findings of the phase-3 trials by Brown et al. (8) and Mahajan et al. (7), we have initiated the ESTRON trial (NCT03285932, currently recruiting) to examine the potential of fractionated post-operative cavity radiosurgery (24). It randomizes FSRT using the 7 × 5 Gy regimen discussed above with a generous margin of 4 mm and including the surgical tract, against post-operative WBRT. Additionally, neurocognition is assessed in detail during follow-up with the help of validated test batteries.

Limitations of this analysis include its retrospective design with inherent selection bias, as well as the relatively small number of patients. Though propensity score-adjustment was performed in multivariate modeling to minimize selection bias and imbalances between treatment groups, the remaining of a residual bias cannot be safely ruled out. This has to be considered especially when interpreting results for OS in subgroup comparison. Detailed information regarding systemic treatments received concurrent to and following radiotherapy was not available for all patients and could thus not be adjusted for in the analysis. With modern substances rapidly gaining relevance to the prognosis of BM, future analyses will have to be adjusted for this potential confounder.

To the best of our knowledge, this is the first adequately sized single-center analysis to perform a propensity score-adjusted head-to-head comparison of SRS/FSRT and WBRT following the resection of brain metastases. Our study is strengthened by the similarity between WBRT and SRS subgroup sizes, which allows for detailed evaluation of prognostic factors, as well as a dedicated subgroup analysis to identify relevant treatment-related parameters. Local control following stereotactic treatment compares favorably to data found in literature; presumably the use of margins up to 4 mm and fractionated dose-regimens for a relevant number of patients had a beneficial effect. The results of our analysis demonstrate that despite available phase-3 data for the post-operative radiotherapy of BM, several relevant questions remain yet to be answered in a prospective setting, particularly regarding target definition, radiation dose and fractionation.

## Conclusion

In this propensity score-adjusted head-to-head comparison of SRS/FSRT and WBRT following the resection of brain metastases, patients who received stereotactic treatment showed longer OS and LC compared to patients treated with WBRT, and LC compared favorably to recent literature. We found the extent of macroscopic resection to be the strongest prognostic factor across subgroups for local control and survival. Additional favorable prognosticators included good clinical performance and better RPA and GPA scores; unfavorable prognosticators included lung cancer histology. Future analyses will address the ideal choice of safety margin, dose and fractionation for postoperative stereotactic RT of the resection cavity.

## Data Availability Statement

The datasets generated for this study will not be made publicly available since national legislation and the terms of study ethics approval do not allow dataset sharing outside of the institutions participating in the analysis.

## Ethics Statement

This study was performed following institutional guidelines and the Declaration of Helsinki of 1975 in its most recent version. Ethics approval for the study and a waiver of written informed consent was granted by the Heidelberg University ethics committee on April 12th, 2018 (#S-172/2018). Patient confidentiality was maintained by anonymizing patient data to remove any identifying information.

## Author Contributions

RE, DB, JD, and SR planned and supervised this analysis as part of the neuro-radiooncological research group. TD performed data extraction and review. DW performed all statistical analysis. RE reviewed data analysis and drafted the manuscript. DS, KL, LK, SH, TF, BN, TE, and SA contributed patient data and participated in reviewing and improving analysis and manuscript. All authors read and approved the final manuscript.

## Conflict of Interest

The authors declare that the research was conducted in the absence of any commercial or financial relationships that could be construed as a potential conflict of interest.
